# Craving fullness: a fullness-seeking phenotype that blurs the line between binge eating disorder and food addiction

**DOI:** 10.3389/fpsyt.2026.1848242

**Published:** 2026-05-25

**Authors:** Vera I. Tarman, David A. Wiss

**Affiliations:** 1 Renascent Foundation, Toronto, ON, Canada; 2 Nutrition in Recovery LLC, Los Angeles, CA, United States

**Keywords:** binge eating disorder (BED), compulsive high-volume eating (CHVE), dopamine and reward learning, eating behavior, food addiction (FA), fullness-seeking phenotype, GLP-1 and satiety hormones, gut-brain reward axis

## Abstract

Food addiction and binge eating disorder show striking clinical overlap that current diagnostic frameworks do not fully capture. In binge eating disorder samples, Yale Food Addiction Scale-defined food addiction has been reported in roughly half of participants. Higher symptom severity is associated with greater impairment. Within this overlap, clinicians frequently observe a subset of patients whose compulsive eating is organized around the pursuit of extreme fullness rather than palatability for specific trigger foods. Compulsive high-volume eating (CHVE) refers to recurrent, distressing episodes of consuming dangerously large volumes of food, often to the point of marked gastric distension. In some cases, the food is low-caloric or non-hedonic, and the dominant motivator is the interoceptive target state of extreme fullness rather than taste. This Perspectives article focuses on fullness-seeking as a high-volume pattern within binge eating disorder phenomenology that has received little attention in the food addiction literature. It extends prior work that framed volume addiction within compulsive high-volume eating as a public health issue. The focus here is clinical formulation, not the public health case. Evidence from reward learning, gut-brain signaling, gastrointestinal physiology, and neuroendocrinology suggests that this pattern may be rooted in binge eating disorder. It may identify a subset of patients with addiction-like dynamics. These cases may be better addressed when addiction-informed concepts and tools are integrated. It offers a physiologically grounded lens for combining eating disorder and addiction frameworks in a more coherent clinical approach.

## 1 Introduction: two diagnoses, one clinical reality

Binge eating disorder and food addiction have historically been conceptualized and treated as distinct entities, yet in practice the boundary is porous. Many patients identify with both (1, 2). In binge eating disorder samples, 42–57% meet Yale Food Addiction Scale criteria for food addiction, and this overlapping subgroup shows greater distress and impairment than either group alone (3–5). In this paper, binge eating disorder refers to the formal DSM-5 diagnosis, whereas Yale Food Addiction Scale-defined food addiction refers to an index of addiction-like symptoms that parallel criteria for substance use disorders. Within this overlap, clinicians frequently observe patients whose compulsive high-volume eating episodes are organized around the pursuit of extreme fullness rather than palatability or specific trigger foods.

Compulsive high-volume eating (CHVE) is proposed as a cross-cutting high-volume eating pattern rather than a distinct diagnosis. It can occur within or outside those thresholds and may, in some cases, involve addiction-like dynamics. It is marked by recurrent, distressing episodes of consuming dangerously large volumes of food, in some cases even low-caloric or non-hedonic foods (6). The emphasis here is not that CHVE is categorically separate from binge eating disorder, but that some CHVE presentations appear to be especially organized around reaching an interoceptive endpoint of extreme fullness. In these presentations, the reinforcing pull may be less tied to specific highly palatable foods and more tied to the internal state produced by volume and gastric distension. A growing literature calls for broader integration of eating disorder and food addiction frameworks (1, 7–9). Recent work by Ifland and by Wiss and colleagues is especially relevant here because it argues that addictive eating and eating disorder models should be brought into closer dialogue (7–9). This Perspective extends that agenda by presenting compulsive high-volume eating as a proposed gut-brain-reward pattern within CHVE that links satiety physiology, nutritional volume, and addiction-like dynamics across binge eating disorder symptomatology.

CHVE remains understudied as a distinct phenomenon within eating pathology (6). A recent paper by Tarman framed volume addiction as a public health issue and argued that compulsive high-volume eating warrants recognition as a meaningful eating pattern (6). The present paper extends rather than replicates that work. It examines how the overlap between eating disorder and food addiction models may sharpen clinical understanding, assessment, and treatment of CHVE. Prevalence estimates are not yet available because compulsive high-volume eating has not been uniquely or consistently operationalized in either the eating disorder or food addiction literature. At present, this fullness-seeking subset within CHVE remains clinically derived. It is not yet specified well enough to support standardized screening or case definition. Still, recurring clinical observation of patients who seek fullness primarily, together with converging indirect evidence for a volume addiction profile, supports defining this phenomenon more clearly and studying it systematically. In Werdell’s clinical work, these descriptions draw on decades of treating individuals with food addiction and volume addiction in intensive settings, though they remain based on clinical observation rather than controlled trials (10).

Among high-volume eaters, the dominant motivator may be the internal sensation of extreme fullness rather than taste or a specific forbidden food. These individuals may describe a drive to feel maximally sated and to achieve relief, calm, completion, or safety (11). In this framing, food volume becomes the vehicle for reaching a target interoceptive state. Some report consuming large amounts of high-bulk foods or fluids until they feel tight, pressured, and finally calm. Others describe an escalating need for greater volume to reach the same internal endpoint. They may also feel irritable or agitated when they try to stop short of maximal fullness. This fullness-seeking pattern may represent a CHVE configuration described across binge eating disorder and food addiction presentations.

The central argument is not that CHVE is better characterized as binge eating disorder or food addiction, but that it can contain clinically meaningful features of both. It often resembles binge eating disorder because it includes loss of control with food, distress, and functional impairment, even if the motivating reinforcer is not primarily hedonic (12, 13). At the same time, it may include addiction-like features such as tolerance-driven escalation in food volume over time and withdrawal-like distress when prevented from reaching a usual level of fullness. These observations are consistent with addiction-like features described in binge eating disorder more generally (14) and with practice-based reports from abstinence-oriented food addiction programs where volume eating is explicitly identified as an addictive pattern (6, 8).

This article draws on reward learning, gut-brain, endocrine, and stress literatures to argue for an integrative framework. The aim is to show why CHVE may be usefully conceptualized at the intersection of binge eating disorder and food addiction, and to highlight broad directions for research and integrated, mechanism-matched clinical care (1, 15). This Perspective is hypothesis-generating rather than systematic.

## 2 Shared biology: a unified model for BED, FA, and CHVE

This section highlights three domains where compulsive high-volume eating illustrates convergence between binge eating disorder and food addiction: reward learning and dopaminergic wanting versus liking, gut-brain satiety signaling, and stress-linked modulation of relief-seeking. The mechanisms discussed below are intended as plausible and hypothesis-generating. They are not presented as established explanations specific to CHVE.

### 2.1 The dopamine connection

Understanding how dopamine neurobiology influences overeating may help explain core features of both binge eating disorder and food addiction and may also be relevant to the fullness-seeking aspect of compulsive high-volume eating. Dopamine is a key signal in mesolimbic reward circuits that marks cues, actions, or internal states as worth pursuing (15, 16). When episodes of overeating reliably reduce negative affect, reward circuits may learn to treat that pattern as important and repeatable (11). As a result, urges to overeat can persist even when the immediate pleasure of the food is modest or, we speculate, when fullness itself becomes aversive but still motivating.

The distinction between wanting and liking helps clarify this clinical picture. Wanting can be high even while liking stays flat or falls (17, 18). People may then feel a strong urge to repeat a behavior that no longer feels good. In compulsive high-volume eating, wanting may shift away from a specific food and toward an internal state, namely the stretched, heavy sensation of maximal fullness. If repeated episodes have paired extreme fullness with relief, calmness, or perceived safety, dopaminergic wanting may become tied to that interoceptive state rather than to the taste of the food. Clinically, patients often describe needing to feel full more than needing a specific food. This could reflect a dopamine-linked pursuit of relief through fullness rather than classic hedonic overeating.

Loss of inhibitory control adds another layer to this shared biology. Weaker top-down frontal braking over reward circuits makes it harder to stop once a behavior has started (19, 20). In compulsive high-volume eating, this can manifest as continued eating well beyond intention or comfort once the fullness sequence is underway. When impaired inhibitory control combines with a learned drive toward fullness as relief-seeking, the result can be both distressing and hard to stop, with escalating volume over time and continued use despite bodily harm. These may represent addiction-like features layered onto a more classic eating disorder presentation (14).

## 3 The moving target of “enough”: interoceptive satiety, endocrine signaling and stress vulnerability

In fullness-seeking compulsive high-volume eating, the key reinforcer may be interoceptive: the internal state of being maximally full rather than the properties of any given food. The biological systems that translate stomach filling into feeling enough include gastric stretch receptors, vagal afferent pathways, and downstream satiety hormones such as GLP-1, PYY, leptin, and ghrelin (21–23). These systems sit at the intersection of satiety physiology and affect regulation, providing a biological network in which eating disorder and addiction frameworks are both relevant (1).

As the stomach distends, mechanosensitive vagal afferents signal this stretch to the brainstem and broader parasympathetic circuitry (22). Increased vagal tone and parasympathetic activation are generally associated with reductions in arousal and anxiety. The interoceptive state of deep fullness may therefore be experienced as calming and become a reinforcing target in compulsive high-volume eating (23).

Animal research suggests that vagal afferent mechanosensitivity is plastic. It is reduced by diet composition, chronic stress, and circadian disruption, so greater gastric distension may be required to achieve the same satiety-linked vagal response and subjective enough endpoint (24). This offers a physiological bridge to the clinical interpretation of tolerance. If the same internal state requires progressively more volume to achieve, escalation becomes predictable. Clinically, this maps onto reports that progressively higher amounts of food are needed to reach the same level of fullness and relief. Gastric stretch signaling via vagal afferents is therefore a plausible route by which volume may acquire reinforcing properties.

Recent work also suggests that the gut-brain-vagal axis interacts with the mesolimbic dopamine reward system. In mice, intact vagal input is required for normal mesolimbic dopamine responses (25). When vagal signaling is disrupted, dopamine cells fire less to palatable food or drugs, dopamine signals in the nucleus accumbens are blunted, and reward-seeking behavior is reduced (25). These findings suggest that vagus-mediated signals from gastric distension may not only convey fullness but may also shape how reinforcing extreme fullness feels in vulnerable individuals.

Human studies also offer indirect support for this possibility. Fullness thresholds and gastric physiology can differ in binge-type conditions, although volume-seeking patients have not yet been specified in research studies. In water load paradigms, individuals with bulimia nervosa and binge eating disorder typically tolerate larger volumes before reporting fullness, and gastric myoelectrical activity differs from that of controls (26–28). These findings do not prove a fullness-seeking mechanism, but they do show that gastric interoception and tolerance to higher volumes can shift with repeated exposures.

Gastrointestinal mechanics may further influence the relief or calmness that patients pursue. Gastric accommodation determines how much volume can be consumed before discomfort occurs and how attainable maximal fullness is during an episode (27, 29). Gastric emptying governs how long distension persists and how quickly nutrients reach the small intestine. This in turn influences satiety hormones such as GLP-1 and PYY, as well as suppression of hunger signals including ghrelin (30). Together, these mechanisms link physiological processes such as volume tolerance and persistence of distension to motivational consequences such as how easily a relief state can be achieved.

Endocrine signals further modulate how increases in gastric volume are translated into feelings of fullness and the will to stop eating. Leptin resistance, which can arise in chronic overeating, diet-induced obesity, and sustained hyperleptinemia, can blunt satiety and alter reward circuitry (13, 21). Ghrelin levels should decline as the stomach fills after a meal, yet in bulimia nervosa, binge eating disorder, and obesity post-meal suppression is relatively blunted, so appetitive signaling remains elevated (30, 31). Together, these dysregulated signals can plausibly contribute to both the desire to reach greater fullness and the sense that this is not enough yet.

GLP-1 pathways are especially relevant because they link gut filling to both satiety and reward (34). GLP-1 receptor agonists influence gastric emptying and act within central motivational circuits, including mesolimbic dopamine pathways (32, 33). Chronic high-fat, high-energy intake and binge-like eating have both been associated with reduced GLP-1 responsiveness, with consequences that may include delayed satiety and weaker post-meal dampening of reward drive (34). Under normal conditions, GLP-1 helps limit both caloric intake and volume by promoting satiety and dampening food reward. In compulsive high-volume eating, reduced responsiveness could weaken these braking signals so that patients escalate volume further before achieving the same sought-after sense of enough (32–34).

Stress physiology adds another bridge because fullness-seeking is often described as relief-seeking rather than pleasure-seeking. Stress and arousal systems can increase binge vulnerability and weaken self-control, and fullness may function as a swift conditioned response to reduce arousal (11, 35, 36). Oxytocin may provide a further link. It has been highlighted in the eating disorder literature as a hormone that helps regulate stress responses and eating behavior, interacting with parasympathetic rest-and-digest states (37). In fullness-seeking compulsive high-volume eating, oxytocin-sensitive vagal and central pathways may help dampen arousal after eating and may converge with vagal and dopaminergic mechanisms to reinforce extreme fullness as a preferred relief state.

Taken together, these strands support studying CHVE as a possible phenomenon in which established binge eating disorder features such as loss of control, distress, and impairment overlap with addiction-like processes described in the broader binge eating disorder literature (1, 14). Because the proposed reinforcer is interoceptive, this presentation is best examined using eating disorder and addiction frameworks alongside gut-brain models of reward, relief, and learning.

## 4 Clinical implications

A single illustrative and hypothetical case highlights why CHVE may require an integrated lens. Maria, a 42-year-old nurse with long-standing anxiety, describes an evening pattern she calls “topping up my tank.” Most nights she eats a normal dinner, then returns to the kitchen to make mammoth bowls of salad, broth-based soup, and air-popped popcorn, sometimes adding several glasses of water. She says the taste is nothing special but feels driven to keep going until her stomach feels heavily full, at which point her racing thoughts subside and she becomes drowsy. When she tries to stop after a standard meal, she feels restless and uncomfortably empty until she eats enough to recreate that deep stretched sensation of fullness. This scenario illustrates a presentation in which volume, fullness, and relief appear to be the primary targets rather than specific palatable foods.

### 4.1 Assessment

If CHVE contains clinically relevant elements of both binge eating disorder and food addiction, then relying on a single framework for assessment and treatment will likely miss key features. A binge eating disorder-informed lens remains essential because it targets loss of control, distress, impairment, shame-driven restriction, and compensatory behaviors (12, 13). At the same time, an addiction-informed lens adds clinically actionable targets adapted from addiction-like formulations of binge eating and relapse dynamics in the broader literature (14). These include cue-triggered craving for fullness, tolerance-like escalation in volume, withdrawal-like distress when stopping short of maximal fullness, continued use despite harm, and relapse when stress or routine disruption triggers a return to the behavior.

This dual framing matters because fullness-seeking compulsive high-volume eating may not respond to interventions focused only on food type or palatability. The aim is not to define a separate diagnosis, but to help clinicians recognize when a high-volume eating presentation is especially organized around fullness as the primary target. Assessment should identify when the dominant target is an internal state of maximal fullness and relief rather than a specific food or taste experience. Clinically, this may be suggested when patients describe chasing a maximally full sensation rather than a preferred taste. It may also be suggested when episodes center on high-bulk or even low-hedonic foods or fluids, or when stopping short of extreme fullness produces marked distress, agitation, or a sense of incompletion. An integrated assessment would ask not only what foods and how much, but also what internal state is being pursued and how that target state has changed over time. It should assess whether fullness-seeking is central, whether tolerance-like escalation in volume is present, and whether attempts to reduce volume are experienced as distressing in clinically meaningful ways (6, 11, 14, 27). Integration does not imply that binge eating disorder treatments ignore these mechanisms, but that an addiction framework makes reward-governed, tolerance-like, and relapse-like dynamics more explicit and provides a shared language for cravings, cues, and recovery-oriented supports (1, 14, 15).

### 4.2 Psychological and behavioral interventions

In practical terms, an integrated platform supports a combination of eating disorder safeguards and structure with addiction-informed behavioral strategies. Eating disorder-informed components might include consistent and balanced eating patterns, reduction of chaotic restriction cycles that amplify stress and hunger signals, psychotherapy approaches that address shame and rigidity, and monitoring for compensatory behaviors (38–40).

Yet eating disorder-informed care alone may leave key maintenance processes in compulsive high-volume eating unaddressed, particularly cue-driven urges to pursue extreme fullness and relapse-like returns to high-volume episodes after periods of control. Addiction-informed components can retain eating disorder safeguards while explicitly naming reward-driven patterns and validating patients’ experience of an addictive pull toward fullness. When fullness is the primary target, treatment may also need to focus explicitly on cues for volume-seeking. It may also need to address tolerance of stopping before maximal fullness and alternative ways of managing agitation, emptiness, or incompletion without escalating volume. Concrete tools may include functional analysis of episodes with attention to internal states such as stress, emptiness, or incompletion. They may also include trigger mapping, craving-management skills, urge-surfing strategies, and relapse-prevention planning (14, 41, 42). For some patients, this can extend to peer-based supports such as 12-step programs, provided these are integrated with eating disorder safeguards and not used to reinforce shame-based restriction or weight-centric goals (43).

Because volume seeking may be central in this presentation, treatment may need to address meal volume and fullness, not only caloric content (6, 26, 27). Behavioral interventions may also need to target portion structure and meal timing more explicitly, using planned meal volume (weighing and measuring), predictable eating intervals, and pacing strategies to reduce escalation toward extreme fullness. This could also include gradual recalibration toward a tolerable level of moderate fullness while avoiding restrictive or punitive control and remaining anchored in established eating disorder safeguards (40).

### 4.3 Pharmacological considerations

Pharmacologic approaches that act on both satiety and reward systems offer an additional rationale for integration (34). GLP-1 receptor agonists are one example. They can act on satiety physiology and reward-linked motivation, although evidence in binge eating disorder and related conditions is still emerging and larger controlled trials are needed (32, 33, 44). Parallel work in addiction medicine suggests that GLP-1 receptor agonists can also reduce alcohol intake and other substance misuse outcomes, supporting the idea that GLP-1 pathways modulate shared reward circuits across overeating and substance use (45).

In CHVE terms, medications that alter gastric emptying and satiety signaling while dampening motivational drive could plausibly reduce the urgency to pursue extreme distension. More broadly, the proposed mechanisms point to treatment potential rather than proof of treatment efficacy. The core clinical implication is that clinicians may need to address both the eating disorder symptoms and the addiction dynamics, or they risk prioritizing either the psychosocial or the biological when both need to be integrated (1, 8, 12).

## 5 Conclusion and future directions

By foregrounding extreme fullness as the primary reinforcer, the fullness-seeking CHVE pattern within overlapping binge eating disorder and food addiction presentations may make the intersection between these frameworks more visible and clinically useful. Patients like Maria illustrate how this could appear in practice. The pattern combines binge eating disorder-like loss of control and distress with addiction-like escalation, withdrawal-like distress, persistence despite harm, and relapse-like cycling organized around extreme fullness (6, 14). The argument is not that binge eating disorder and food addiction are interchangeable. It is that CHVE may require both frameworks: binge eating disorder-informed approaches to address classic eating disorder phenomenology and risk, and addiction-informed approaches to target escalation and relapse dynamics when extreme fullness becomes the target state (1, 39, 41). For now, this is best understood not as a separate diagnosis, but as a clinically observed fullness-seeking configuration within CHVE that warrants clearer operationalization and prospective study.

Future work should aim to make CHVE clinically and scientifically measurable without prematurely treating it as a formal diagnosis. A first step would be to develop a clearer operational definition of compulsive high-volume eating that includes fullness-seeking drive, tolerance-related escalation, and withdrawal-associated distress as candidate features of a fullness-driven pattern. A second priority is prospective phenotyping across eating disorder, obesity, and addiction-focused settings. This would help clarify how common this presentation is, how it relates to binge eating disorder and Yale Food Addiction Scale-defined food addiction, and which clinical trajectories and risks it carries (6, 46).

A third priority is mechanistic research focused on the gut-brain axis. This could combine volume-tolerance paradigms and gastric physiology with endocrine measures including ghrelin, leptin, and GLP-1 pathways (23, 27). It could also incorporate stress-linked vulnerability. Future work should also clarify how this fullness-seeking configuration overlaps with other binge-type presentations, including bulimia nervosa. It should also clarify overlap with other compulsive presentations without presuming diagnostic equivalence. Qualitative studies of patients who describe this pattern could help characterize lived experience and refine future interventions (47). Finally, pharmacology and mechanistic studies should stratify participants by compulsive high-volume eating features and predefine relevant endpoints. This would allow treatments to be evaluated for their effects on the proposed fullness-reinforcement loop rather than only on weight or general appetite (32, 44). Nutritional interventions that manipulate meal portions, structure, energy density, and fluid versus solid volume should also be explored as experimental levers (6, 23).

If these steps are taken, CHVE can move from a recurring but under-measured clinical observation to a better-characterized phenomenon that exemplifies when and how eating disorder and food addiction frameworks should be integrated. In turn, this may open the door to more precise, compassionate, and biologically informed care for patients who live in bodies where feeling full is still not enough.

## Data Availability

The original contributions presented in the study are included in the article/supplementary material. Further inquiries can be directed to the corresponding author.
